# ROP18 Is a Key Factor Responsible for Virulence Difference between *Toxoplasma gondii* and *Neospora caninum*

**DOI:** 10.1371/journal.pone.0099744

**Published:** 2014-06-13

**Authors:** Tao Lei, Hui Wang, Jing Liu, Huizhu Nan, Qun Liu

**Affiliations:** Key Laboratory of Animal Epidemiology and Zoonosis, Ministry of Agriculture, and National Animal Protozoa Laboratory, College of Veterinary Medicine, China Agricultural University, Beijing, China; University at Buffalo, United States of America

## Abstract

*Toxoplasma gondii* (*T. gondii*) and *Neospora caninum* (*N. caninum*) are both obligate intracellular protozoan parasites and share many common morphological and biological features. Despite these similarities the two parasites differ dramatically in virulence in mice, but the factors involved in virulence differences between the two parasites remain unknown. A secreted serine-threonine kinase called rhoptry protein 18 (ROP18) was identified to play a crucial role on virulence differences among different *T. gondii* clonal lineages. Intriguingly, we found that ROP18 in Nc1 strain of *N. caninum* (NcROP18) is a pseudogene due to several interrupting stop codons in the sequence in our previous studies. We assume that the difference of ROP18 leads to virulence difference between *T. gondii* and *N. caninum.* We constructed a transgenic *N. caninum* Nc1 stain by transfecting the TgROP18 from the *T. gondii* RH strain. Phenotype and virulence assays showed that the expression of TgROP18 in *N. caninum* did not affect the motility and cell invasion, but resulted in a significant increase in intracellular parasite proliferation and virulence in mice. Immunity-Related GTPase (IRG) phosphorylation assay showed that the transgenic parasite Nc1-TgROP18 was able to phosphorylate IRGs as *T. gondii* did. The present study indicated that the ROP18 plays a crucial role in virulence of the closely related parasites *T. gondii* and *N. caninum* and it is indeed a key factor responsible for the virulence difference between *T. gondii* and *N. caninum*.

## Introduction

*Toxoplasma gondii* (*T. gondii*) and *Neospora caninum* (*N. caninum*) are closely related protozoan parasites of the phylum Apicomplexa [1]. They are both obligate intracellular parasites that cause a wide range of diseases in different host species. They also share many common morphological and biological features, such as developing in intermediate hosts, reproducing asexually, or to move between intermediate and definitive hosts, reproducing sexually [2]. Because of the similarities between the two parasites, *N. caninu*m was initially misidentified as *Toxoplasma* [3]–[4].

Despite these similarities the two parasites differ dramatically in virulence in experimental animals. The RH wild-type strain of *T. gondii* causes lethal infection in all strains of laboratory mice with one tachyzoite (LD_100_≈1) [5]–[6], whereas the Nc1 wild-type strain of *N. caninum* is much less virulent with a median lethal dose (LD_50_) 10^7^ tachyzoites or higher (unpublished data). A secreted serine-threonine kinase called rhoptry protein 18 of *T. gondii* (TgROP18), which can bind to and phosphorylate immunity-related GTPases (IRGs) [7], was identified as the key virulence factor of *T. gondii* [5]. In previous studies, we found that ROP18 in Nc1 strain of *N. caninum* (NcROP18) is a pseudogene due to several interrupting stop codons in the sequence, which was confirmed by the findings of Reid et al [4].

We suspected that ROP18 might be responsible for the virulence difference between *T. gondii* and *N. caninum*. In order to test this hypothesis, we constructed the Nc1 strain of *N. caninum* stably expressing the TgROP18 gene using pyrimethamine-resistant DHFR-TS and green fluorescent protein (GFP) genes as double-selection markers, and evaluated the phenotypes and virulence of the transgenic parasite.

## Materials and Methods

### Ethics statement

All experiments with animals in this study were performed in strict accordance with the recommendations in the Guide for the Care and Use of Laboratory Animals of the Ministry of Science and Technology of China. All experimental procedures were approved by the Institutional Animal Care and Use Committee of China Agricultural University (The certificate of Beijing Laboratory Animal employee, ID: 18049). All efforts were made to minimize animal suffering.

### Parasite culture and preparation

The *N. caninum* Nc1 wide-type strain, the *T. gondii* RH wide-type strain and the Nc1-GFP strain (kindly provided by Professor Xuenan Xuan, Obihiro University of Agriculture and Veterinary Medicine, Japan), which is a transgenic parasite by transfecting Nc1 wild-type strain with the pDMG plasmid and expressing green fluorescent protein (GFP), were propagated as tachyzoites by serial passages in human foreskin fibroblast (HFF) cell as previously described [8]. Briefly parasites were cultured in the Dulbecco's Modification of Eagle's Medium (DMEM) (pH 7.4) supplemented with L-glutamine, 10% heat-inactivated fetal bovine serum (FBS), penicillin (100 U/mL) and streptomycin (100 ug/mL) at 37°C and 5% CO_2_. Parasites were harvested and isolated by washing in cold phosphate-buffered saline (PBS), centrifugation, resuspension in cold PBS, syringing three times through a 27-gauge needle, filtering through a 5.0 µm pore filter (Millipore, USA), washing twice with PBS, and finally centrifugation at 2,000 rpm for 10 min [9].

### Construction of the transfer vector pDMG-TgROP18

The genomic DNA was extracted from *T. gondii* RH tachyzoites with phenol-chloroform and precipitated with ethanol [10]. A synonymous mutation at the *EcoRV* site of the TgROP18 gene (GenBank: JX045330) except the stop codon was obtained by PCR amplification of two overlapping sections (1,243 bp and 465 bp respectively) using the following primer pairs: F1+R2 and F2+R1. Primer sequences were: F1 5′-cgGATATCATGTTTTCGGTACAGCGG-3′, R1 5′-ccgATGCATTTCTGTGTGGAGATGTTC-3′, F2 5′-GTTCAAGCTCAGGGAATT-GTGCATACGGACATTAAACCGGCGAATT-3′, R2 5′-AATTCGCCGGTTTAATGTCCGT-ATGCACAATTCCCTGAGCTTGAAC-3′. Primers F1 and R1 were introduced to the *EcoRV* and *NsiI* sites (underlined), respectively. After purification, the PCR product was double digested with *EcoRV* and *NsiI* (NEB, USA). Then the recovered fragment was inserted into the pDMG vector (kindly provided by Professor Xuenan Xuan, Obihiro University of Agriculture and Veterinary Medicine, Japan), which is a transfer vector for constructing recombinant *T. gondii* expressing foreign genes [11]–[12]. The resulting plasmid was designated as pDMG-TgROP18. The TgROP18 gene was fused with the reporter gene GFP, and the fused TgROP18-GFP gene is under the control of *T. gondii* GRA1 promoter. The pDMG-TgROP18 was used as a transfer vector to transfect *N. caninum*.

### Transfection and selection of *N. caninum* stably expressing TgROP18

Transfection of *N. caninum* was carried out by electroporation as described previously [12]–[13]. Freshly lysed-out *N. caninum* Nc1 tachyzoites were washed and resuspended at 2–5×10^7^/ml with 50 µg of pDMG-TgROP18 in a cytomix buffer (120 mM KCl, 0.15 mM CaCl_2_,10 mM K_2_HPO_4_-KH_2_PO_4_, 25 mM Hepes, 2 mM EDTA, 5 mM MgCl_2_, pH 7.6) supplemented with 2 mM ATP and 5 mM glutathione. The parasites were transferred to a 0.2 cm gap cuvette and electroporated with 2 kV at 25 µFd and 50 Ω with the Gene Pulser Xcell electroporation system (BioRad, USA). After electroporation, the parasites were allowed to recover for 15 min at room temperature before inoculation to HFF cells grown in 25 cm^2^ T-flasks. Recombinant parasites were selected on HFF cells in the presence of pyrimethamine at a concentration of 1 µM. After 10 generations of selection, the pyrimethamine-resistant and fluorescent parasites were isolated by flow cytometry (Beckerman MoFlo XDP, USA), and the isolated recombinant parasite stably expressing TgROP18 was designated as Nc1-TgROP18.

### Production of recombinant TgROP18 and Irgb6 protein and of Anti-TgROP18 and Anti-Irgb6 serum

The expression of TgROP18 and Irgb6 protein as the (His)_6_-tag fusion protein in *Escherichia coli* (*E. coli*) and production of Anti-TgROP18 and Anti-Irgb6 serum in BALB/c mice and rabbits were carried out as described previously [14]. The open reading frame of TgROP18 without the stop codon was amplified by PCR using primers introduced to the *BamHI* and *XhoI* sites (underlined): 5′-cgGGATCCATGTTTTCGGTACAGCGG-3′ and 5′-ccgCTCGAGTTCTGTGTGGAGATGTTC-3′. Total RNA was extracted from Ana-1 cells (kindly provided by Dr Xiangmei Zhou, China Agricultural University), which is a murine macrophage cell line, with the Trizol reagent (Invitrogen, USA), and cDNA was synthesized by using EasyScript First-Strand cDNA Synthesis SuperMix kit (TransGen, China). The cDNA was used as the template for Irgb6 cloning. The open reading frame of Irgb6 without the stop codon was amplified by PCR using primers introduced to the BamHI and XhoI sites (underlined): 5′-cgGGATCCATGGCTTGGGCCTCCAGCTTTGA-3′ and 5′-ccgCTCGAGA-GCTTCCCAGTACTCGGGGGGCT-3′. The PCR products were inserted into the expression vector pET-28a (Novagen, Germany) and then incorporated into *E. coli* for protein expression. The recombinant proteins fused to a (His)_6_-tag were expressed and purified using HisTrap FF purification columns (Novagen, Germany) as described by the manufacturer. Female specific-pathogen-free (SPF) BALB/c mice aged 6 weeks old and New Zealand white rabbits were purchased from the Laboratory Animal Center of Academy of Military Medical Sciences (Beijing, China). For the first injection, mice were immunized subcutaneously with 100 µg of purified recombinant TgROP18 or Irgb6 protein in an equal volume of Freund's complete adjuvant (Sigma, USA), while rabbits were immunized subcutaneously with 1 mg of purified recombinant TgROP18 or Irgb6 protein in an equal volume of Freund's complete adjuvant. The second and third injections were carried out in 2 and 4 weeks post-primary injection with 50 µg (mice) or 500 µg (rabbits) of the antigen in Freund's incomplete adjuvant (Sigma, USA). Anti-TgROP18 and Anti-Irgb6 serums were collected 2 weeks after the last immunization.

### Determination of TgROP18 expression in Nc1-TgROP18

#### Immunofluorescence assay (IFA)

The recombinant *N. caninum* stably expressing TgROP18 isolated by flow cytometry was firstly confirmed by IFA as previously described [15]. Briefly, the parasites were fixed with 4% (v/v) paraformaldehyde (PFA) and permeabilized with 0.2% Triton-100 in PBS for 20 min, and then blocked with PBS supplemented with 3% BSA for 30 min at room temperature. The samples were incubated for 60 min with mouse Anti-TgROP18 serum diluted at 1∶100 in 1% BSA-PBS as the primary antibodies, washed and incubated for 60 min with Texas Red-conjugated Anti-mouse IgG (Proteintech, USA) as the secondary antibody. Finally, the parasites were observed under a laser confocal scanning microscope (Leica TCS SP5 II, Germany).

#### PCR

The genomic DNA was extracted from the *N. caninum* Nc1 strain, Nc1-GFP strain, transgenic Nc1-TgROP18 strain, and *T. gondii* RH strain. PCR detection was performed using specific primers for TgROP18 (primer sequences: 5′-ATGTTTTGGTACAGCGG-3′ and 5′-TTCTGTGTGGAGATGTTC-3′) and GFP (primer sequences: 5′-ATGCATAAAGGAGA-AGAAC-3′ and 5′-TTATTTGTATAGTTCATCCAT-3′).

#### Quantitative real-time PCR (qRT-PCR)

Total RNA was extracted from the tachyzoites of RH wide-type strain and transgenic Nc1-TgROP18 strain with the Trizol reagent. cDNA was synthesized by using EasyScript First-Strand cDNA Synthesis SuperMix kit (TransGen, China). The 18sRNA gene was selected as the endogenous reference gene. qRT-PCR was performed using specific primers for TgROP18 (primer sequences: 5′-TGAGAAGGCGGAT-TCTGGATG-3′ and 5′-CCTTAACAGCCAACTCTTCATTGGTCT-3′) and 18sRNA (primer sequences: 5′-ATTAGATACAGAACCAACCCAC-3′ and 5′-TGAATGATCCGTCGCAGAC-3′).

#### Western blot

Total lysates were prepared from purified Nc1, Nc1-GFP, Nc1-TgROP18 and *T. gondii* RH tachyzoites and subjected to sodium dodecyl sulfate-polyacrylamide gel electrophoresis as described previously [15]. Following electrophoresis, separated proteins were transferred onto polyvinylidene fluoride (PVDF) membranes (Millipore, USA). The PVDF membrane was blocked with 5% (w/v) skim milk diluted in PBS for 60 min at room temperature before incubation with mouse Anti-TgROP18 serum (dilution 1∶500) and rabbit Anti-GFP polyclonal antibody (dilution 1∶1,000; Proteintech, USA) on a separate membrane. After washing in PBST, the PVDF membrane was incubated with goat Anti-mouse IgG horseradish peroxidase (HRP)-labelled secondary antibody (dilution 1∶10,000; Proteintech, USA) or goat Anti-rabbit IgG HRP-labelled secondary antibody (dilution 1∶10,000; Proteintech, USA). Labelled proteins were visualized with ECL chemiluminescence reagents (CoWin Biotech, China).

### Plaque assay

The plaque assay was performed as described previously [16]. Monolayers of HFF grown in 6-well plates were infected with tachyzoites and incubated for 7 days at 37°C. The cells were fixed with 4% PFA for 20 min, and then stained with Crystal violet for 10 min and washed with water. Plaques were visualized under the microscope (4× objective). The Nc1 wide-type strain incubated for 30 min at 50°C was used as the negative control.

### Transmigration assay

Freshly egressed parasites were added to the upper compartment of the Transwell system (Corning, USA) containing 200 µL ringer buffer (155 mM NaCl, 3 mM KCl, 2 mM CaCl_2_, 1 mM MgCl_2_, 3 mM NaH_2_PO_4_, 10 mM Hepes, 10 mM Glucose, pH 7.4). The lower compartment below the membrane (3 µm pore) contained 500 µL of the same solution. The parasites were incubated at 37°C in a CO_2_ incubator and samples for counting from the lower compartment were taken at the indicated times.

### Gliding motility assay

The gliding motility assay was carried out as described previously [17]–[18]. Eight-well glass chamber slides were coated overnight at 4°C with 50% fetal bovine serum in PBS (pH7.4) and wash slides with PBS before use. Freshly lysed parasites were filtered, pelleted, and resuspended in HHE (Hank's Balanced Salt, 10 mM HEPES, 1 mM EGTA) and allowed to glide on previously FBS coated slides at 37°C for 15 min. Parasites were fixed with 4% PFA and IFA using the Anti-TgSAG1 antibody (RH strain) or Anti-NcSRS2 antibody (Nc1, Nc1-GFP and Nc1-TgROP18 strains) was performed to visualize the trails.

### Cell invasion assay

Cell invasion assays were performed as described previously [19]. Freshly egressed parasites were inoculated on HFF cells seeded on 24-well plates for 30 min. The extracellular parasites were removed by washing three times with PBS. The cells were further incubated for 24 h before fixation for IFA (Nc1 and RH strains) or visualization under a fluorescence microscope (Nc1-GFP and Nc1-TgROP18 strains). The number of vacuoles representing successful invasion events was counted. The Nc1 strain incubated for 30 min at 50°C before use as the negative control. Three independent experiments were performed.

### Intracellular growth assay

For intracellular growth assays, freshly lysed parasites were inoculated on cells seeded on 24-well plates for 30 min. The extracellular parasites were removed by washing three times with PBS and the resulting intracellular parasites allowed to grow for 24 h before fixation for IFA (Nc1 and RH strains) or visualization under the fluorescence microscope (Nc1-GFP and Nc1-TgROP18 strains). The rate of intracellular growth was monitored by counting the number of parasites per vacuole. The parasites of at least 100 vacuoles were counted for each condition, and the results are representative of three independent experiments.

### Virulence assay in mice

Female BALB/c mice of 6 weeks old were purchased from the Laboratory Animal Center of Academy of Military Medical Sciences (Beijing, China). They were housed under specific pathogen-free conditions for 7 days before manipulation. Food and water were freely available throughout the experiments. The tachyzoites of Nc1, Nc1-GFP, Nc1-TgROP18 and RH strains grown in HFF cells monolayers were purified from freshly lysed HFF cells. The tachyzoites were injected intraperitoneally (IP) mice at 10, 10^3^ or 10^6^ per animal. All animals were monitored three times a day for clinical signs and mortality for 30 days post injection. The mice were humanely euthanized when they were unable to reach food or water for more than 24 h and lost 20% normal body weight. All mice were monitored three times a day (every 8 hours). The mice were humanely euthanized by cervical dislocation after anesthetization. The mice were anesthetized by subcutaneous injection of Atropine (0.02 mg/kg) before euthanasia.

### IRG phosphorylation assay

IRG phosphorylation assay was performed by IFA on Ana-1 cells. Briefly, IFNγ-stimulated Ana-1 cells were seeded on coverslips and infected with freshly harvested parasites. At 12 h after challenge, coverslips were fixed for IFA. The following immunoreagents were used: mouse Anti-TgROP18 serum (dilution 1∶100), Anti-Irga6 phosphopeptide Ab T102-555 (1∶1000) (kindly provided by Professor J.C. Howard, University of Cologne, Germany), fluorescein isothiocyanate (FITC)-conjugated goat Anti-mouse antibody (dilution 1∶100; Proteintech, USA) and Texas Red-conjugated goat Anti-rabbit antibody (dilution 1∶100; Proteintech, USA). Finally, the parasites were observed under a laser confocal scanning microscope.

### Statistical analysis

Statistical calculations were performed as described previously [20]. P values were calculated in Excel using the Student's t-test assuming equal variance, unpaired samples, and using 2-tailed distribution, where P≤0.005 was considered significant, and where P≤0.001 was considered extremely significant. Means and standard deviations (SD) were also calculated in Excel.

## Results

### Construction of the transgenic parasite Nc1-TgROP18

**Figure 1:**
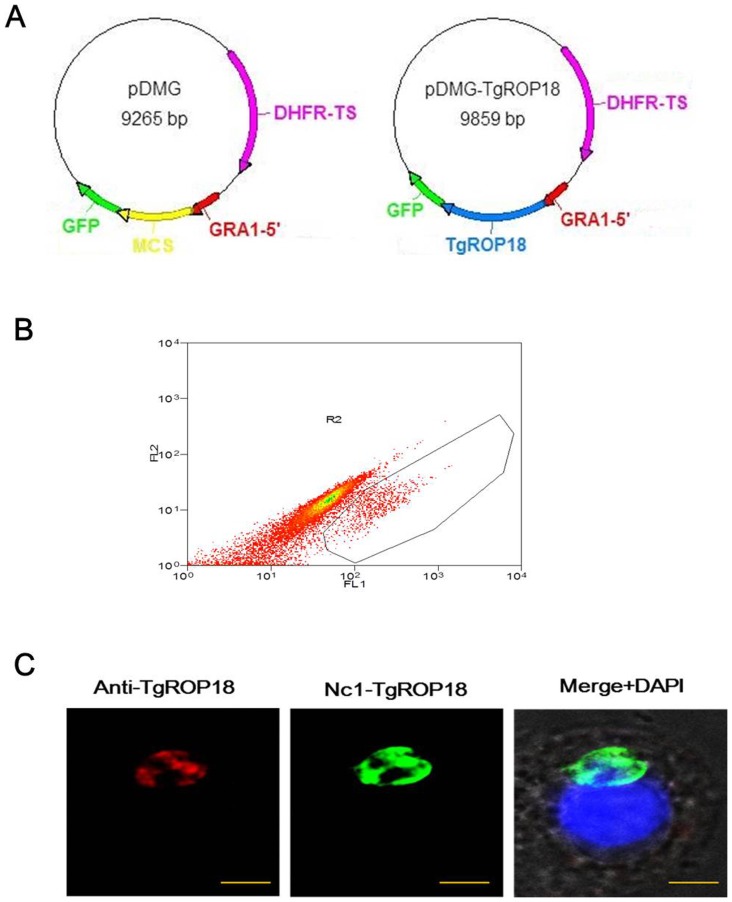
Construction of Nc1-TgROP18. (A) Plasmid map of transfer vectors pDMG and pDMG-TgROP18, respectively. DHFR, dihydrofolate reductase-thymidylate synthase; MCS, multiple cloning site; GFP, green fluorescent protein. (B) The recombinant *N. caninum* stably expressing TgROP18 was isolated by flow cytometry. (C) IFA Localization of TgROP18 in Nc1-TgROP18. The recombinant *N. caninum* stably expressing TgROP18 was confirmed by IFA using mouse anti-rTgROP18 serum as the primary antibody. Scale bar, 5 µm.

To generate *N. caninum* stably expressing the TgROP18 gene, the transfer vector pDMG-TgROP18 carrying the pyrimethamine-resistant DHFR-TS gene, the TgROP18 gene and the reporter GFP gene in tandem was first constructed (Fig. 1A). The tachyzoites of Nc1 were transfected with pDMG-TgROP18 by electroporation, and the drug-resistant recombinant parasites with bright green fluorescence were observed under the fluorescence microscope 24 h after transfection. After 10 generations of selection by pyrimethamine, a recombinant *N. caninum* stably expressing TgROP18 was isolated by flow cytometry (Fig. 1B) and designated as Nc1-TgROP18.

### Identification of Nc1-TgROP18

**Figure 2:**
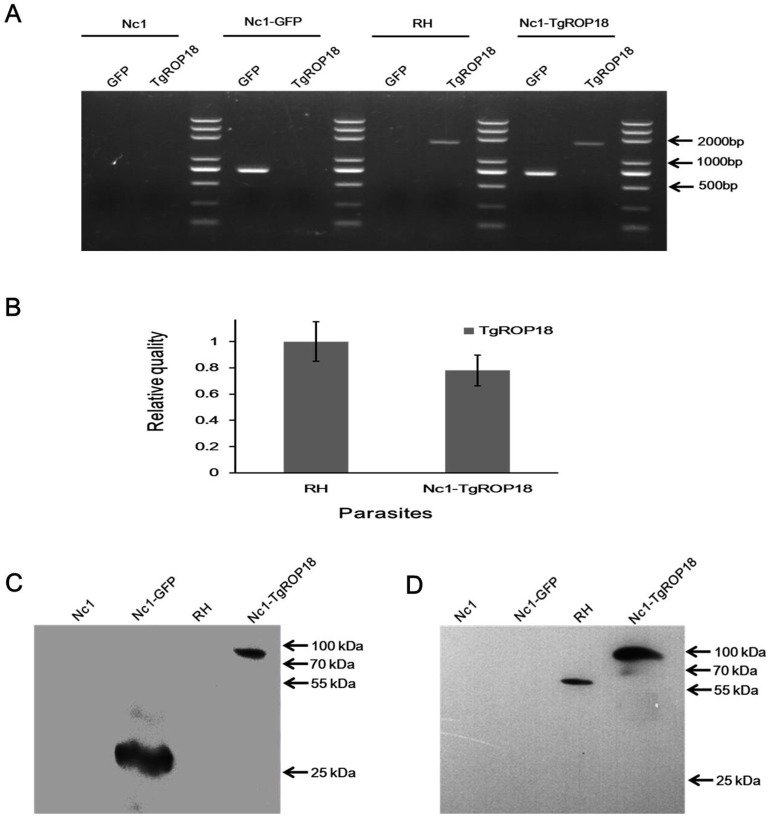
Identification of Nc1-TgROP18. (A) Electrophoresis of the PCR products. Both the GFP and TgROP18 target genes (717 bp and 1,662 bp, respectively) were amplified from the genomic DNA of the Nc1-TgROP18 strain. (B) qRT-PCR comparison of TgROP18 expression in RH strain and Nc1-TgROP18 strain. Data are mean ± SD (error bars) of three independent experiments. (C) and (D) Expression of TgROP18 in the cloned parasites was confirmed by Western blot analysis. The TgROP18 and GFP fusion protein (∼87 kDa) was recognized by both anti-GFP and anti-TgROP18 polyclonal antibodies.

The expression of TgROP18 in the isolated parasites was confirmed by IFA, PCR, qRT-PCR, and western blot. When analyzed by IFA, TgROP18 was found at the apical end of Nc1-TgROP18 tachyzoites, colocalizing exactly with GFP, which was similar to that observed for *T. gondii* and is consistent with localization in rhoptries (Fig. 1C). Electrophoresis of the PCR products showed that both the GFP and TgROP18 target genes (717 bp and 1,662 bp, respectively) were amplified from the genomic DNA of the Nc1-TgROP18 strain, while only the GFP target gene was amplified from the genomic DNA of the Nc1-GFP strain, the TgROP18 target gene from the genomic DNA of RH strain, and neither gene from the genomic DNA of the Nc1 wild-type strain (Fig. 2A). As shown in Fig. 2B, transgene of TgROP18 in the Nc1-TgROP18 strain was expressed just a little below the RH wide-type strain (not statistically significant) on the mRNA level. Western blot analysis confirmed the expression of both GFP and TgROP18. The TgROP18 and GFP fusion protein (∼87 kDa) expressed by the Nc1-TgROP18 strain was recognized by both Anti-TgROP18 and Anti-GFP polyclonal antibodies, while only the TgROP18 protein (∼60 kDa) was detected with polyclonal antibodies against TgROP18 in RH strain, and only the GFP protein (∼27 kDa) was detected with polyclonal antibodies against GFP in the Nc1-GFP strain (Fig. 2C). Collectively, these results demonstrated that the transgenic parasite Nc1-TgROP18 was successfully constructed.

### Phenotype assays of Nc1-TgROP18

**Figure 3:**
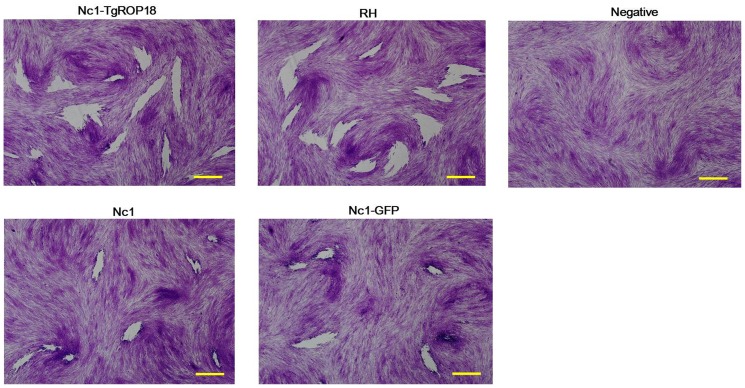
Plaque assay. The indicated strains grew on HFF cells for 7 days before fixation and staining with Crystal violet. The Nc1 strain incubated for 30°C was used as the negative control. Three independent experiments were performed and results of one representative experiment are shown here. Scale bar, 200 µm.

Plaque formation measures parasite survival and replication in cell culture, and reflects parasite motility on the surface of the host cell layer, invasion, intracellular growth and egress [21]. Any change of these phenotypes of the transgenic Nc1-TgROP18 can be determined by the plaque assay. Seven days after inoculation of HFF cells, the transgenic Nc1-TgROP18 strain formed plaques of similar size to the *T. gondii* RH strain, but much bigger than those of the *N. caninum* Nc1 and Nc1-GFP strains (Fig. 3), indicating that at least one of the steps in the lytic cycles of the Nc1-TgROP18 strain was increased compared to the Nc1 wild-type strain and the expression of TgROP18 in Nc1 strain was most likely responsible for the change.

**Figure 4:**
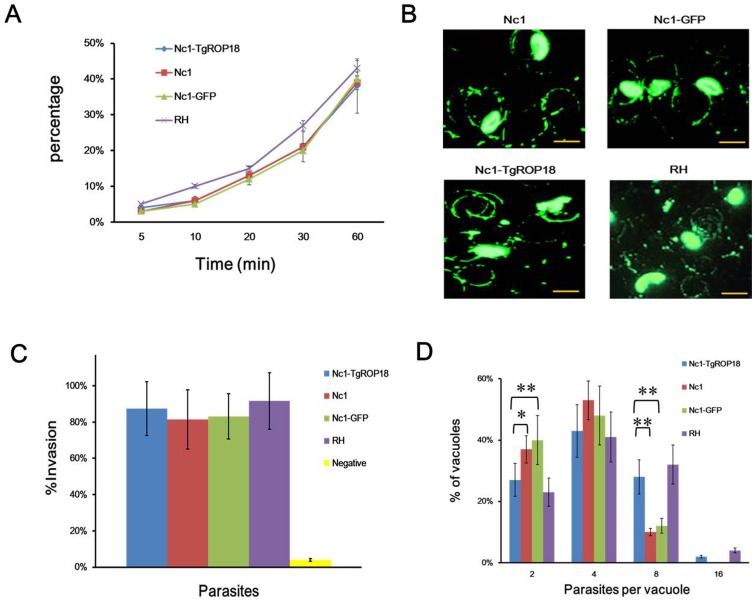
Phenotype assays of Nc1-TgROP18. (A) Transmigration assay in the Transwell system. Data are represented as mean ± SD (error bars) of three independent experiments. (B) Gliding motility assay. The trails were stained with the anti-TgSAG1 antibody (RH strain) or anti-NcSRS2 antibody (Nc1, Nc1-GFP and Nc1-TgROP18 strains). The arrow indicates a trail. Scale bar, 5 µm. (C) Cell invasion assay showed no difference in cell invasion between the transgenic Nc1-TgROP18 strain and the untransfected Nc1 or *T. gondii* RH strain. Data are mean ± SD (error bars) of three independent experiments. (D) Intracellular replication assay of the transgenic Nc1-TgROP18 compared to the Nc1 strain, Nc1-GFP strain and the RH strain. Asterisks indicate statistically significant results (*P≤0.005; **P≤0.001), as determined with the Student's t test. Data are mean ± SD (error bars) of three independent experiments.

More detailed analyses were undertaken to determine which step has changed. Transmigration assays using the Transwell system showed that the motility of the Nc1-TgROP18 strain was comparable with that of the Nc1 and Nc1-GFP strain, but slightly below the RH strain (not statistically significant) (Fig. 4A), suggesting transfection of Nc1 with TgROP18 did not affect its motility *in vitro*, which was further confirmed by the finding of the gliding motility assay (Fig. 4B). Similar results were obtained when cell invasion assays were performed, in which the transgenic Nc1-TgROP18 strain showed no alteration of invasion compared with the other three strains (Fig. 4C). Then intracellular replication assays were carried out to establish whether stably expressing TgROP18 in Nc1 strain improved the ability of the parasite to proliferate in host cells. Parasites that invaded host cells 24 h after inoculation were analyzed for intracellular growth by counting the number of parasites per vacuole. Because of the lack of synchronization of host cell invasion, the intracellular vacuoles contained 2, 4, 8, or 16 parasites. As shown in Fig. 4C, the distribution of 4 parasites per vacuole did not significantly differ between the four strains. In contrast, the number of the vacuoles containing 2 parasites of the transgenic Nc1-TgROP18 strain was significantly lower than that for the Nc1 wide-type strain and the Nc1-GFP strain, while the rate of the vacuoles containing 8 parasites of the transgenic Nc1-TgROP18 strain was significantly higher than that for the Nc1 wide-type strain and the Nc1-GFP strain. Moreover, a small percentage of vacuoles containing 16 parasites, which was absent in the Nc1 and Nc1-GFP strains, were observed in the transgenic Nc1-TgROP18 strain. There was no statistically significant difference in intracellular replication between the Nc1-TgROP18 strain and the *T. gondii* RH strain. These findings indicated that TgROP18 expression in *N. caninum* led to a significant increase in parasite proliferation, and suggested that ROP18 was probably responsible for the intracellular proliferation of *T. gondii* and *N. caninum*.

### Virulence assay in mice

**Figure 5:**
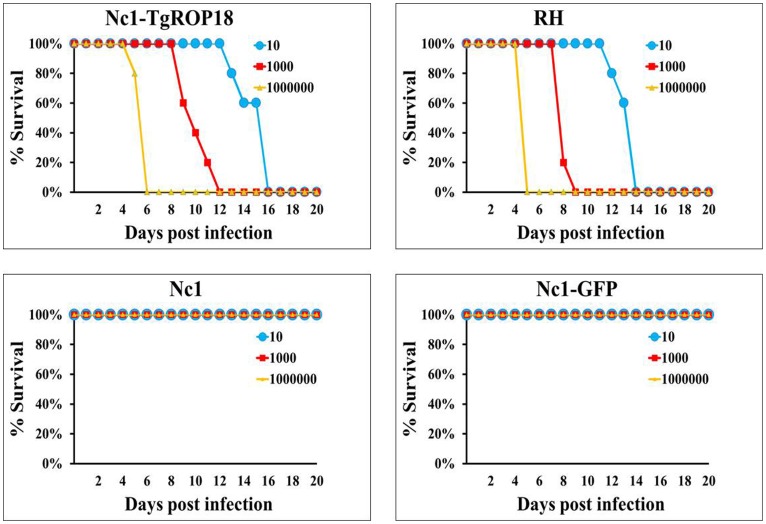
Virulence assay in mice. Mice (n = 5) were injected with 10, 10^3^ or 10^6^ tachyzoites and were monitored for 30 days. All mice injected with the Nc1-TgROP18 strain and *T. gondii* RH strain died within 16 days post infection, compared with no death of mice infected with Nc1 wide-type or Nc1-GFP tachyzoites. Three independent experiments were performed and one representative is shown here.

Since the results above demonstrated that expression of TgROP18 in *N. caninum* led to increased intracellular parasite proliferation, the mouse infection assay was then performed to assess the contribution of TgROP18 to virulence of the transgenic parasite. Mice were inoculated IP with the transgenic Nc1-TgROP18 strain, Nc1 wide-type strain, Nc1-GFP strain and RH wide-type strain at 10, 10^3^ or 10^6^ tachyzoites per mouse. All mice injected with tachyzoites of the Nc1-TgROP18 strain died within 5∼16 days post infection, and the mortality kinetics was similar to that observed in mice inoculated the RH strain. In contrast, there was no death of the mice infected with Nc1 wide-type and Nc1-GFP tachyzoites at any dose level in 30 days after infection (Fig. 5). The data showed that transfection of Nc1 with TgROP18 enhanced dramatically the virulence of Nc1 compared to the parental Nc1 wide-type, suggesting that TgROP18 was responsible for parasite proliferation.

### IRG phosphorylation assay

**Figure 6:**
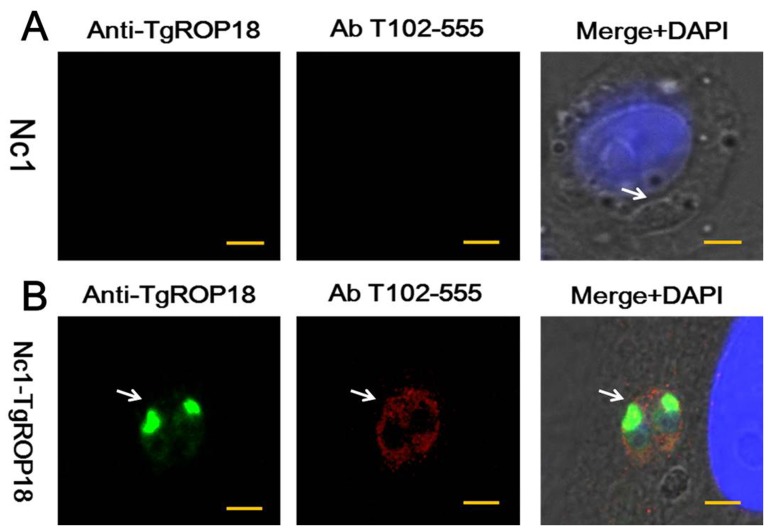
IRG phosphorylation assay. (A) and (B) Localization of phosphorylated Irga6 in IFNγ-stimulated Ana-1 cells infected with Nc1 wide-type strain or transgenic Nc1-TgROP18 strain, respectively. TgROP18 localized with mouse Anti-TgROP18 serum (FITC, green), Irga6 localized with rabbit Anti-Irga6 phosphopeptide Ab T102-555 (Texas Red, red), and nuclei stained with DAPI (blue). Scale bar, 2.5 µm.

To determine whether transgenic parasite Nc1-TgROP18 is able to phosphorylate IRGs we examined the recruitment of IRGs to the parasitophorous vacuole (PV) based on the protein Irga6, which is treated as one of the most important indicator of the host IRGs family and implicated in resistance to *T. gondii*. We observed that only Nc1-TgROP18 was able to phosphorylate Irga6 (Fig. 6A, B), indicating the transgenic parasite can inactivate the IRGs due to the transgenic expression of TgROP18.

## Discussion

*T. gondii* and *N. caninum* are closely related protozoan parasites, but the two parasites differ greatly in virulence. The factors involved in virulence differences between the two parasites have not been investigated. In this study, our findings identified ROP18 as a key factor responsible for virulence difference between *T. gondii* and *N. caninum* for the first time.

*T. gondii* is an obligate intracellular parasite belonging to the phylum of Apicomplexa, which includes a great number of important human and animal pathogens such as *Cryptosporidium*, *Eimeria*, *Neospora*, *Plasmodium*, and *Theileria* [21]–[22]. Because of its importance as an opportunistic pathogen and experimental advantages on genetic manipulation in the laboratory, *T. gondii* has emerged as a major model for the study of intracellular parasitism [23]. *N. caninum* is also an obligate intracellular protozoan parasite [24], which is known to have morphological and biological characteristics highly similar to *T. gondii*. Because of the similarities between the two parasites, *N. caninum* was initially misidentified as *T. gondii* for many years [3]–[4]. The use of *N. caninum* as a heterologous system for the expression of genes from *T. gondii* has been explored, and it may prove to be useful for the identification of parasite factors that are involved in the phenotypic differences between these two closely-related parasites [25]–[26], which was confirmed by transgenic expression of TgROP18 from *T. gondii RH* strain in *N. caninum* Nc1 strain in the present study.

Most strains of *T. gondii* belong to one of three distinct clonal lineages, referred to as type I, type II and type III [27]–[28]. Despite having less than 2% genetic diversity, the three *Toxoplasma* lineages differ significantly in virulence among other differences. Type I strains are the most virulent and cause mortality at doses as low as one parasite (LD_100_≈1) [29], whereas type II and type III have median lethal doses in mice ranging from 10^2^ to 10^5^ (LD_5_≈ 10^2^∼10^5^) [30]–[31]. Forward genetic analysis accompanied by quantitative trait locus (QTL) mapping revealed that TgROP18, a highly polymorphic serine-threonine kinase, which secretes from a specialized apical organelle named rhoptries during host cell invasion [32]–[33], and is subsequently delivered to the cytosolic side of the PV, plays a crucial role on virulence differences among *T. gondii* clonal lineages. TgROP18 differs dramatically in expression among the three clonal lineages of *T. gondii*, and more exactly type III express 1,000-fold lower levels compared to type I and type II [5]–[6]. Transgenic expression of TgROP18 from RH strain, a type I lineage, in the avirulent type III CTG strain causes a four log increase in virulence in the mouse model [5]. Consistent with this, overexpression of TgROP18 in type I strain results in a significant increase in intracellular parasite proliferation rate, which is closely related to virulence [22]. In contrast, clean deletion of TgROP18 in the virulent type I strain leads to virulence attenuation [34]–[35].

*N. caninum* is much less virulent in mice even compared to the avirulent type III strain of *T. gondii*, but factors responsible for the virulence difference between the two closely-related parasites remain unknown. TgROP16, which is a tyrosine kinase that directly phosphorylates the host signal transducer and activator of transcription 3 (STAT3) and STAT6 [36]–[37], was identified as another key virulence factor of *T. gondii*, but it may be not responsible for virulence difference between *T. gondii* and *N. caninum*, due to the fact that ROP16 of *N. caninum* (NcROP18) is also able to phosphorylate STAT family of proteins (unpublished data). Intriguingly, we found that NcROP18 is a pseudogene due to several interrupting stop codons in the sequence by accident in our previous studies, which was confirmed by the findings of Reid AJ [4]. Therefore, we suspected that the difference of the ROP18 gene leads to virulence difference between *T. gondii* and *N. caninum*. Thus a transgenic parasite was constructed by transfecting the TgROP18 of RH strain into *N. caninum* Nc1 stain. The phenotypes and virulence assays show that transgenic expression of TgROP18 in *N. caninum* does not affect its motility and invasion, but leads to a dramatical increase in intracellular parasite proliferation rate and virulence in mice.

*T. gondii* is capable of infecting essentially any warm-blooded animals by actively invading nucleated host cells and forming PV [38], which provides a protective niche for the parasite to avoid immune clearance and promote intracellular proliferation. A family of immunity-related GTPases (IRGs) plays an important role in resistance to *T. gondii* and a lot of other intracellular pathogens, including *Chlamydia*, *Mycobacteria*, *Leishmania*, *Listeria*, and *Salmonella* [39]–[40]. For *T. gondii*, recruitment of IRGs on the PVM leads to its disruption, thereby resulting in parasite degradation [41]–[42]. The serine-threonine kinase TgROP18 of virulent type I strain is able to phosphorylate IRGs on key threonine residues in switch region I of the GTPase domain, which results in block IRG recruitment and protects the parasite from attack by the mouse immune system [43]–[44]. *N. caninum* as well as type II and type III strains of *T. gondii* are unable to inactivate the IRGs because of active ROP deficient, which was confirmed by our observations in the present study [45]–[46]. Recent studies have revealed that a pseudokinase named TgROP5 was required for the catalytic activity of the active TgROP18 in type I strain, and both the two proteins were necessary to avoid IRG recruitment on PVM of *T. gondii* [20]. Transfection of the virulent ROP18 allele into avirulent type III strain enhanced parasite growth and caused a four log increase in virulence in the mouse model [5], indicating that TgROP5 performs similar function in both virulent type I strain and avirulent type III strain of *T. gondii*. Therefore, we speculated ROP5 of *N. caninum* (NcROP5) could regulate the activity of TgROP18 based on the results of the present study that the transgenic parasite Nc1-TgROP18 was able to phosphorylate IRGs as *T. gondii* did. Another possibility is that a novel and undiscovered *N. caninum* rhoptry protein plays the same role as TgROP5 [4].

In conclusion, the present study confirmed that TgROP18 plays a crucial role on virulence of *T. gondii* from a different angle by expression of *T. gondii* genes in *N.caninum*. More significantly is that this is the first study to investigate what is responsible for the virulence difference between *T. gondii* and *N. caninum*, and our findings finally identify ROP18 is indeed the key factor of virulence difference between the two closely related protozoan parasites.
